# FRET-Based Detection and Quantification of HIV-1 Virion Maturation

**DOI:** 10.3389/fmicb.2021.647452

**Published:** 2021-03-09

**Authors:** Anamaria D. Sarca, Luca Sardo, Hirofumi Fukuda, Hiroyuki Matsui, Kotaro Shirakawa, Kazuki Horikawa, Akifumi Takaori-Kondo, Taisuke Izumi

**Affiliations:** ^1^Department of Hematology and Oncology, Graduate School of Medicine, Kyoto University, Kyoto, Japan; ^2^Department of Biological Sciences, University of the Sciences, Philadelphia, PA, United States; ^3^Department of Optical Imaging, Advanced Research Promotion Center, Tokushima University, Tokushima, Japan

**Keywords:** HIV-1 Gag maturation, Förster Resonance Energy Transfer, single virion imaging, protease inhibitor, fluorescence microscopy

## Abstract

HIV-1 infectivity is achieved through virion maturation. Virus particles undergo structural changes via cleavage of the Gag polyprotein mediated by the viral protease, causing the transition from an uninfectious to an infectious status. The majority of proviruses in people living with HIV-1 treated with combination antiretroviral therapy are defective with large internal deletions. Defective proviral DNA frequently preserves intact sequences capable of expressing viral structural proteins to form virus-like particles whose maturation status is an important factor for chronic antigen-mediated immune stimulation and inflammation. Thus, novel methods to study the maturation capability of defective virus particles are needed to characterize their immunogenicity. To build a quantitative tool to study virion maturation *in vitro*, we developed a novel single virion visualization technique based on fluorescence resonance energy transfer (FRET). We inserted an optimized intramolecular CFP-YPF FRET donor-acceptor pair bridged with an HIV-1 protease cleavage sequence between the Gag MA-CA domains. This system allowed us to microscopically distinguish mature and immature virions via their FRET signal when the FRET donor and acceptor proteins were separated by the viral protease during maturation. We found that approximately 80% of the FRET labeled virus particles were mature with equivalent infectivity to wild type. The proportion of immature virions was increased by treatment of virus producer cells with a protease inhibitor in a dose-dependent manner, which corresponded to a relative decrease in infectivity. Potential areas of application for this tool are assessing maturation efficiency in different cell type settings of intact or deficient proviral DNA integrated cells. We believe that this FRET-based single-virion imaging platform will facilitate estimating the impact on the immune system of both extracellular intact and defective viruses by quantifying the Gag maturation status.

## Introduction

While the acquired immunodeficiency syndrome (AIDS) is a deadly disease caused by infection with human immunodeficiency virus type 1 (HIV-1), AIDS-related deaths have been reduced due to the tremendous efforts that have gone into researching the virus itself and ways to counteract it (Centers for Disease Control and Prevention (CDC), 2006). Combination antiretroviral therapies (cART) significantly decrease AIDS mortality and reduce further transmission of HIV-1 (Castilla et al., 2005; Kitahata et al., 2009). However, while cART effectively achieves viral suppression and prevents the progression to AIDS, virus eradication or functional cure strategies have not been established yet, and thus lifelong treatments are still required (Holkmann Olsen et al., 2007; Kousignian et al., 2008). The major obstacle to achieving a cure for HIV-1 is the existence of latently infected reservoir cells within memory CD4 T cells and macrophages that can persist even during cART (Chun et al., 1997; Finzi et al., 1997; Siliciano et al., 2003; Hassan et al., 2016; Wong et al., 2019). Latent HIV-1 persistent reservoirs are established early in the acute phase of infection (Finzi et al., 1997, 1999; Daar et al., 1998; Zhang et al., 2000; Whitney et al., 2014; Henrich et al., 2017; Colby et al., 2018). Defective proviruses with sequence deletions and mutations rapidly accumulate within a few weeks after virus infection and persist for decades during the chronic phase (Bruner et al., 2016). The defective proviruses are generated by error-prone reverse transcription, recombination, and other mutation-inducing events such as APOBEC3G mediated G-to-A mutations (Ho et al., 2013; Bruner et al., 2016). Though it was initially thought to have little involvement in HIV-1 pathogenesis, novel unspliced viral RNA transcription was lately identified in defective proviruses which frequently encoded competent gag or gag-pol open reading frames (Ho et al., 2013; Imamichi et al., 2016). In addition, HIV-1 Gag protein expression in cells harboring defective proviruses was detected by fluorescence microscopy (Imamichi et al., 2020). Since most defective proviruses preserve the 5′ end of intact proviral sequences encoding gag and gag-pol (Ho et al., 2013; Imamichi et al., 2016; Hiener et al., 2017), they may be able to assemble and release virus-like particles into the extracellular space.

Viral maturation is the final step of the HIV-1 life cycle and crucial for the formation of infectious virions (Freed, 2015). The structural Gag polyprotein is cleaved into the matrix (MA), capsid (CA), nucleocapsid (NC), and p6 proteins in a stepwise manner by the viral protease (Mattei et al., 2018). The CA protein assembles to form a mature viral core that houses the viral genome, nucleocapsid, reverse transcriptase, and integrase and stabilizes the lipid bilayer of the virus particle (Davidoff et al., 2012; Pornillos and Ganser-Pornillos, 2019). After viral membrane fusion to enter the target cell, the core protects the viral genome from host sensor proteins such as cGAS, serves as location for reverse transcription, and traffics the pre-integration complex as far as the integration site (Forshey et al., 2002; Gao et al., 2013; Rankovic et al., 2017; Novikova et al., 2019; Siddiqui et al., 2019; Burdick et al., 2020). In addition to its role in the HIV-1 life cycle, virion maturation may also play an important role in the ability of the virus to escape immune responses. In this regard, it has been reported that maturation of defective viral particles induces strong cellular responses, such as IFN-γ production, T cell stimulation, and B cell mediated antibody production through efficient Env presentation (Alvarez-Fernandez et al., 2012; Gonelli et al., 2019).

Fluorescence microscopy techniques in the field of virology have recently evolved to perform quantitative unbiased analysis based on the development of automated image data processing tools. However, the resolution of fluorescence microscopy is not sufficient to determine the morphological transitions of the viral architecture. Förster Resonance Energy Transfer (FRET) is a principle that relies on the partial spectral overlap of fluorescent protein pairs distanced within 10 nm from each other. Excitation of the donor fluorophore leads to an energy transfer to the acceptor fluorophore, and the emission from the excited acceptor fluorophore is detected (Sekar and Periasamy, 2003). The application of FRET in virology enabled us to visualize the cleavage of HIV-1 Gag by the viral protease (De Rocquigny et al., 2014; Muller et al., 2014; Sood et al., 2017). FRET protein pairs have been optimized to achieve maximum energy transfer, photostability, brightness, and low spectral crosstalk (Bajar et al., 2016). Cyan and yellow fluorescent proteins (CFP and YFP, respectively) are common FRET pairs that allowed long-term time-lapse imaging of live cells (Heim and Tsien, 1996; Kremers et al., 2006). ECFPΔC11 and cp173Venus, derived from CFP and YFP respectively, are a pair that has been developed and used specifically for intramolecular high-intensity FRET in live cells and even *in vivo* (Nagai et al., 2004; Chiu and Yang, 2012).

This study developed a molecular tool to detect and quantify the frequency of immature virions by FRET-based fluorescence microscopy. We achieved this by inserting the ECFPΔC11-cp173Venus FRET pair into the Gag polyprotein between the MA and CA domains with viral protease cleavage sites, to label infectious virions based on the HIV Gag-iGFP construct (Hubner et al., 2007). This new fluorescence based system, that we named HIV-1 Gag-iFRET, showed equivalent infectivity to wild-type viruses, and proportions of immature virions comparable to previous and our Electron Microscopy (EM) analyses (Burdick et al., 2020; Link et al., 2020). We also applied this tool to evaluate HIV-1 protease inhibitor activity by assessing virus maturation and infectivity. We believe that this would also be a useful tool to quantify the maturation of extracellular defective virus particles derived from full-length Gag-Pol coding sequences and to estimate their potential immunogenicity.

## Materials and Methods

### Plasmid Construction

Double-stranded DNA of the intra-molecular FRET pair genes ECFPΔC11 and cp173Venus (Nagai et al., 2004), flanked by HIV-1 protease cleavage sites (AA: SQNYPIVQ, NA: TCGCAGAACTATCCAATTGTACAA) and containing the 3′ end of HIV-1 5′ LTR, HIV-1 Gag MA and the 5′ end of HIV-1 Gag CA domain sequences was synthesized (Supplementary Table 1) and cloned into the pUC57 plasmid (GenScript). The synthesized FRET DNA and pHIV Gag-iGFP (Hubner et al., 2007) plasmids were digested with *Bss*HII and *Sph*I restriction enzymes (New England Biolabs, Inc), purified with the QIAquick Gel Extraction Kit (QIAGEN), and ligated by T4 DNA ligase (New England Biolabs, Inc) to obtain the pHIV-1 Gag-iFRET plasmid. The protease defective mutant HIV-1 Gag iFRETΔPro, was generated by replacing the DNA region in pHIV-1 Gag-iFRET digested by *Sph*I and *Sbf*I with the extracted fragment of the previously reported protease defective NL4-3 construct, pNL-Hc (Adachi et al., 1991).

### Cell Cultures and Virus Production

Adherent HEK293T and TZM-bl cells were cultured in Dulbecco’s Modified Eagle’s Medium (Nacalai Tesque) containing 10% Fetal Bovine Serum and 1% Penicillin Streptomycin Glutamine (Invitrogen) (D10) at 37°C with 5% CO_2_.

FRET labeled virions were produced by co-transfecting HEK293T cells (3.5 × 10^6^ cells/10 cm dish) with the pHIV-1 Gag-iFRET or iFRETΔPro together with the pNL4-3 or pNL4-3ΔPro parental plasmid respectively at a 1:1, 1:10, or 1:20 ratio using a polyethylenimine transfection reagent (GE Healthcare). The culture medium was replaced with fresh D10 with or without Darunavir (Sigma Aldrich) at a final concentration of 0.1, 1.0, 10, 20, 500, or 1000 nM 3.5 h after transfection. The virus-containing supernatant was harvested 24 h after the medium change, filtered through 0.45 μm pore size sterile polyvinylidene difluoride (PVDF, Millipore) membrane, and concentrated up to 20-fold by ultracentrifugation through a 20% sucrose cushion at 25,000 rpm (112,499 *g*) for 90 min at 4°C (CP65; Hitachi Koki Co., Ltd.). The virus pellet was resuspended in 500 μl Hank’s Balanced Salt Solution (HBSS) (–) without phenol red (Wako).

### Single-Virion Imaging Analysis

To visualize the HIV-1 Gag-iFRET/iFRETΔPro labeled virions, the concentrated virus supernatant was 800x diluted in 0.22 μm PVDF filtered Hank’s Balanced Salt Solution (HBSS) (–) without Phenol Red (Wako) and loaded (360 μl) into non-coated 8-well glass-bottom chamber slides (Matsunami), then incubated overnight at 4°C.

Single-virion images were acquired with an A1R MP+ Multiphoton Confocal Microscope (Nikon). Two sets of 21 images were automatically taken for each sample under perfect focus conditions. The first set of images was taken using a 457.9 nm wavelength laser for cyan fluorescent protein (CFP) excitation and by reading the emission spectrums through 482 nm/35 nm or 540 nm/30 nm filter cubes to detect CFP or yellow fluorescent protein (YFP) signals, respectively (FRET images). The second set of images was taken using the 514.5 nm wavelength laser for Venus excitation and by reading the emission spectrum through the 540 nm/30 nm filter cube to detect the YFP signal. The maturation status was defined as FRET efficiency compared with the signal detected in HIV-1 Gag-iFRETΔPro labeled virions.

All images were captured as RAW ND2 datasets and exported to TIFF format files using NIS-Elements (Nikon). Binary images were generated based on the Venus signal to obtain the XY coordinates of each particle. Based on these coordinates, the FRET signal intensity of each virion was extracted from the raw data, and the FRET ratio was calculated for every particle (YFP/[YFP + CFP]) (Preus and Wilhelmsson, 2012). Histograms of distribution were generated for the FRET ratio values within a 100 bins division. Gaussian distribution and Kernel density estimation curves were plotted against the histograms. The proportion of the total Gaussian distribution or Kernel density estimation area overlapped with the HIV-1 Gag-iFRETΔPro area was determined as the proportion of immature virions. The process of image data analysis was performed using an in-house MATLAB program (Fukuda et al., 2019).

### Immunoblotting

Transfected HEK293T cells were lysed using RIPA buffer (Wako) supplemented with 1 mM cOmplete^TM^ protease inhibitor cocktail (Sigma-Aldrich), and the supernatants were used for immunoblotting. Briefly, cells were incubated in the lysis buffer for 15 min at 4°C and then centrifuged at 25,000 *g* for 15 min at 4°C. The pellet was sonicated at 45% output (Ultrasonic Processor, GE50) until completely disrupted (∼10 s), centrifuged again as described above, and then supernatants were collected (cell lysate). The protein concentration was measured by BCA assay (Nacalai Tesque). The SDS-PAGE samples were prepared by mixing the cell lysate with 5x Laemmli buffer [312.5mM Tris-HCl (pH 6.8), 10% Glycerol, 10% SDS] containing 5% β-mercaptoethanol and 4% bromophenol blue, and denaturated at 95°C for 5 min. Virus lysates were also prepared in the same way as cell lysates using virions concentrated as described above. Polyacrylamide gel electrophoresis and protein transfer to PVDF membranes (Immobilon, Millipore) were followed by hybridization with primary antibodies. Blots were probed with either mouse anti-p24 (Abcam, ab9071) or mouse anti-GFP (Thermo Fisher Scientific, MA5-15256) primary antibodies overnight at 4°C. HRP-conjugated anti-mouse IgG antibody (GE Healthcare) was used as a secondary antibody. Immunoblotting images were obtained using the ImageQuant^TM^ LAS 500 system (GE Healthcare). After the initial images were taken, the membranes were incubated for 30 min at 50°C in stripping buffer [62.5 mM Tris-HCl (pH 6.8), 2% SDS, 0.7% β-mercaptoethanol], re-blocked and re-blotted with mouse anti-β-actin primary antibodies as described above.

### Single-Round Infection Assay

The pseudotyped HIV-1 Gag-iFRET labeled virus was produced by co-transfecting HEK293T cells with pHIV-1 Gag-iFRETΔEnv and pNL4-3ΔEnv parental plasmids at three different ratios as described in section “Cell Cultures and Virus Production,” together with the HIV-1 envelope expression plasmid, pSVIII-92HT593.1. The pSVIII-92HT593.1 construct was obtained from Dr. Beatrice Hahn through the NIH AIDS Reagent Program, Division of AIDS, NIAID, NIH: HIV-1 92HT593.1 gp160 Expression Vector (cat# 3077) (Gao et al., 1996). HIV-1 Gag-iFRETΔPro labeled virus was produced by co-transfecting HEK293T cells with pHIV-1 Gag-iFRETΔPro and pNL4-3ΔPro parental plasmid at the same ratios as HIV-1 Gag-iFRET. The viral titer was measured by HIV Type 1 p24 Antigen ELISA (ZeptoMetrix). The following day after 5 × 10^3^ TZM-bl cells seeded in 96 well plates, an equal amount of virus (total of 5 ng HIV-1 p24) was added to the TZM-bl target cells, and then cultured at 37°C for 48 h in a CO_2_ incubator. Luciferase activity in the infected cells was measured with the Luciferase Assay System (Promega) on a 2030 ARVO X3 plate reader (Perkin Elmer) to quantify virus infectivity.

### Transmission Electron Microscopy Images

HIV-1 Gag-iFRET, -iFRETΔPro, or NL4-3 virions were produced as described in section “Cell Cultures and Virus Production” up to the viral pellet. The pellet was then fixed overnight at 4°C with a 4% paraformaldehyde, 2.5% glutaraldehyde in 0.1M PBS solution. The next day, the pellet was washed twice with 0.1M PBS and post-fixed in 1% Osmium tetroxide (OsO4) for 1 h at room temperature (RT), then dehydrated in a series of graded ethanol solutions. After immersion in propylene oxide (Nacalai Tesque), samples were once again immersed in a mixture (1:1) of propylene oxide and LUVEAK-812 (Nacalai Tesque) overnight, embedded in Epon812 resin according to the inverted beam capsule procedure, and polymerized at 60°C for 2 days. Ultrathin sections were examined with an H-7650 electron microscope (Hitachi).

## Results

### Construction of FRET Labeled HIV-1 Virus Particles

**FIGURE 1:**
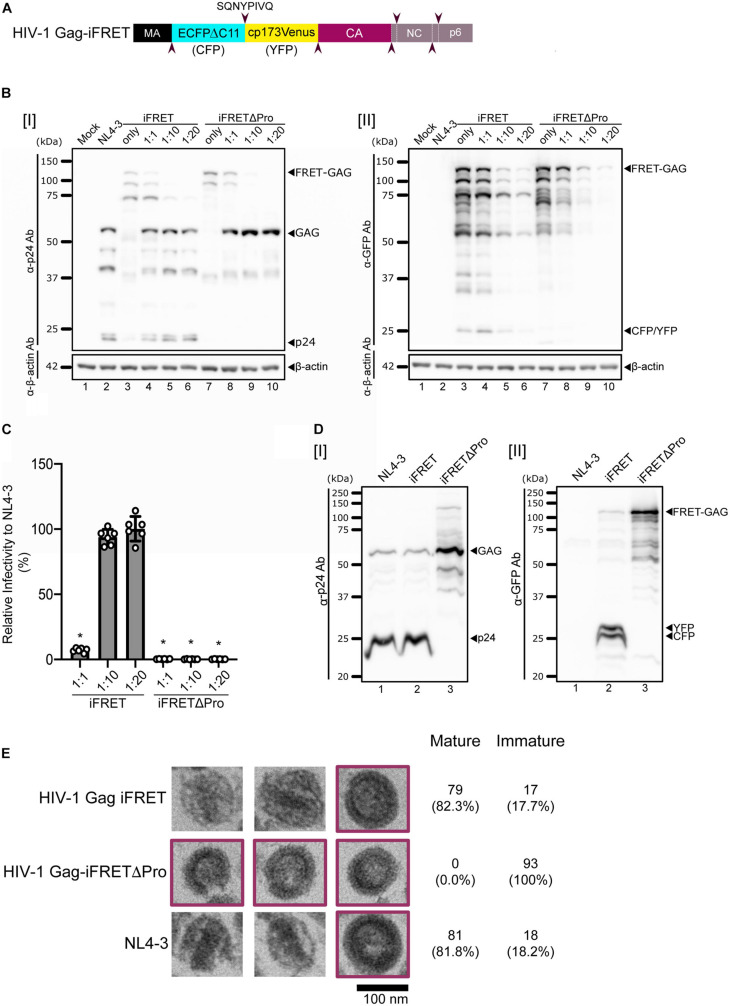
Design and validation of the HIV-1 Gag-iFRET construct. **(A)** Schematic representation of the HIV-1 Gag-iFRET construct in the Gag region. HIV-1 Gag-iFRET was constructed by inserting the efficient single-molecule FRET pair ECFPΔC11-cp173Venus (CFP-YFP) into HIV-1 Gag with HIV-1 protease cleavage sites (SQNYPIVQ, marked by arrowheads). When CFP and YFP are within 10 nm of each other, the excitation energy of the donor CFP transfers to the acceptor YFP and YFP’s emission spectra is detected in the immature virion (uncleaved Gag polyprotein). Once Gag is cleaved by the viral protease and rearranged in the mature virion, the energy transfer efficiency drops, and the FRET signal diminishes. **(B)** Immunoblotting results of cell lysates from HEK293T cells transfected with pNL4-3ΔEnv: pHIV-1 Gag-iFRETΔEnv or pNL4-3ΔPro: pHIV-1 Gag-iFRETΔPro at the indicated ratios, blotted with **[I]** anti-p24 or **[II]** anti-GFP antibodies. The membranes were subsequently stripped and re-blotted with anti-β-actin antibodies. **(C)** Single round infectivity assay using HIV-1 Env-pseudotyped HIV-1 Gag-iFRET and -iFRETΔPro labeled virus produced at the same ratios as in **(B)** was performed in TZM-bl cells. Results are shown as relative infectivity (%) compared to parental NL4-3 virus infectivity. Error bars indicate standard deviation of six independent experiments. Statistical significance was calculated by Wilcoxon matched-pairs signed rank test compared to parental NL4-3 virus infectivity (*p < 0.05). **(D)** Immunoblotting results of virus lysates produced at the 1:10 ratio blotted with **[I]** anti-p24 or **[II]** anti-GFP antibodies. **(E)** Three representative images of HIV-1 Gag-iFRET (top), -iFRETΔPro (middle), and NL4-3 (bottom) virions taken by Transmission Electron Microscopy (TEM). Images of immature virions are highlighted in purple color frames. The numbers of analyzed immature and mature particles together with proportion (%) in brackets are indicated.

Previous studies have shown that inserting a fluorescent protein between the matrix (MA) and capsid (CA) domains of HIV-1 Gag, which is eventually cleaved away during Gag processing by the HIV-1 protease, is compatible with successful assembly and release of infectious HIV-1 virions (Hubner and Chen, 2006; Hubner et al., 2007). To microscopically visualize the viral core generation, we designed a novel bifunctional HIV-1 labeling system that consists of a tandem of cyan- and yellow-emitting fluorescent protein pair as FRET donor and acceptor and named it HIV-1 Gag-iFRET (Figure 1A). We bridged an optimized intramolecular FRET pair, ECFPΔC11 (CFP) and circularly permutated Venus with a new N-terminus starting at Asp-173 (cp173Venus; YFP) (Nagai et al., 2004) with an HIV-1 protease cleavage site, and inserted them between the MA and CA domains of HIV-1 Gag. We hypothesized there would be an efficient energy transfer from the FRET donor (CFP) to the acceptor (YFP) within uncleaved Gag molecules in immature virions. HIV-1 protease cleaves the Gag polyproteins in newly synthesized progeny virions. Thus, HIV-1 Gag-iFRET was designed so that the FRET pair proteins would also be cleaved from the Gag precursor during the maturation process. As a protease deficient mutant to control our experiments, HIV-1 Gag-iFRETΔPro was constructed to contain the same FRET donor-acceptor sequence but could form only immature particles which were expected to have a high FRET efficiency. HIV-1 Gag-iFRET and -iFRETΔPro labeled viral particles were produced by transfecting HEK293T cells with the pHIV-1 Gag-iFRET or -iFRETΔPro constructs alone or at 1:1, 1:10, or 1:20 ratio with the parental pNL4-3 or pNL4-3ΔPro plasmids, respectively. We detected the FRET-pair-fused Gag polyprotein in both HIV-1 Gag-iFRET and -iFRETΔPro transfected HEK293T cells by immunoblot analyses with anti-p24 or anti-GFP antibodies, respectively (Figure 1B). The processed forms of p24 CA and fluorescent proteins (CFP and YFP) were observed in HIV-1 Gag-iFRET transfected cells (Figure 1B lanes 3–6). The cleavage products of Gag were not detected in cells transfected with the HIV-1 Gag-iFRETΔPro at any of the tested ratios (Figure 1B lanes 7–10). Both pHIV-1 Gag-iFRET and -iFRETΔPro construct transfection without their parental helper plasmids seemed to lead to less efficient viral and fluorescent protein expression in the cells (Figure 1B lanes 3 and 7). To evaluate the infectivity of the labeled virus, we performed single-round infection assays using TZM-bl cells with HIV-1 Env-pseudotyped Gag-iFRET viruses. The FRET labeled viruses produced by co-transfection at the 1:10 or 1:20 ratio showed similar infectivity to unlabeled virus (NL4-3), while viruses at the 1:1 ratio dramatically lost their capacity to infect TZM-bl cells (Figure 1C). Therefore, FRET labeled viruses produced at the 1:10 ratio were used for further experiments. The processing of HIV-1 Gag and fluorescent proteins in wild-type and labeled viruses was confirmed by immunoblotting assays of virus lysates with anti-p24 or anti-GFP antibodies, respectively (Figure 1D). We demonstrated that HIV-1 Gag-iFRET virus particles (produced at the 1:10 ratio) contained a conical-shaped structure of the core similar to that of unlabeled parental NL4-3 virions by using Transmission Electron Microscopy (Figure 1E). All protease defective and some of the wild-type virions showed immature morphology (Figure 1E, purple border). We analyzed approximately one hundred virus particles per condition and observed a similar proportion (∼18%) of immature virions as the FRET and control NL4-3 viruses (17 out of 96 and 18 out of 99 particles, respectively). To summarize, HIV-1 Gag-iFRET labeled viruses produced with wild-type Gag, maintained infectivity and displayed a Gag processing efficiency similar to the parental NL4-3.

### Detection of FRET Labeled HIV-1 Virus Particle Maturation

**FIGURE 2:**
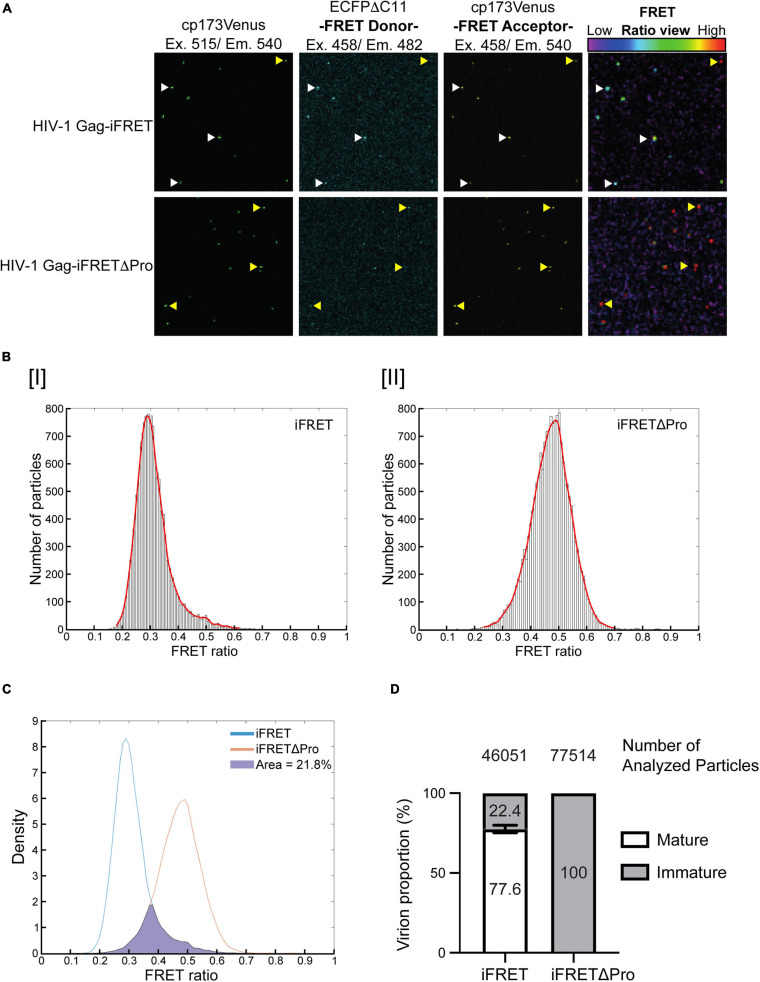
Differentiation and quantification of mature and immature particles in fluorescence microscopy. **(A)** Representative images of FRET labeled virions. All images show the same field containing either HIV-1 Gag-iFRET (top images) or -iFRETΔPro (bottom images) labeled virions. The left panel shows images taken through the YFP excitation (515 nm) and emission (540 nm) channels. The middle panels show images taken by the CFP (FRET donor) excitation (458 nm) and CFP emission (482 nm, left) or YFP emission (540 nm, right) channels. The right panel shows FRET ratio view images that were computationally constructed based on FRET donor (CFP excitation/CFP emission) and acceptor images (CFP excitation/YFP emission) to show FRET efficiency. The color bar indicates that a high FRET signal appears in red (yellow arrows), and the color shifts toward blue (white arrows) as the FRET signal decreases. **(B)** Representative distribution histograms of FRET intensity from **[I]** HIV-1 Gag-iFRET or **[II]** -iFRETΔPro labeled virions are shown with 100 bins. The *x*- and *y*-axis indicate the range of FRET intensity (from 0 to 1) and the number of particles, respectively. The histograms were fitted with a Kernel density estimation function (red curve). **(C)** The Kernel density estimation curves of HIV-1 Gag-iFRET and HIV-1 Gag-iFRETΔPro virions in **(B)** were adjusted to have the same density. The proportion of the HIV-1 Gag-iFRET area under the curve that overlapped that of HIV-1 Gag-iFRETΔPro was calculated and considered as the proportion of immature virions in the total HIV-1 Gag-iFRET virion population. **(D)** Quantification of the mature and immature virion populations based on the calculation strategy in **(C)**. The stacked bar plot shows the average percentage of mature and immature virions in each HIV-1 Gag-iFRET and HIV-1 Gag-iFRETΔPro population. Error bars indicate the standard deviation of three independent experiments. The total number of analyzed particles for each group is shown above their respective graph bar.

Since we confirmed efficient HIV-1 Gag-iFRET and -iFRETΔPro viral particle production with similar Gag processing and infectivity as the parental NL4-3, we next visualized single virions to distinguish their maturation status by quantifying FRET in fluorescence microscopy. A set of FRET images was taken with HIV-1 Gag-iFRET and -iFRETΔPro labeled virions produced at the 1:10 ratio (Figure 2A, upper and lower panels, respectively). Images taken by YFP (cp173Venus) excitation and emission were used to determine the presence of virus particles and their location coordinates for further analysis (Figure 2A, left panels). Representative images taken through the CFP excitation channel and reading the emission of both CFP (FRET Donor) and YFP (FRET Acceptor) are shown (Figure 2A, middle left and right panels, respectively). The ratio view images were constructed based on FRET donor and acceptor images, showing the FRET energy transfer efficiency from donor to acceptor [FRET ratio = YFP emission/(YFP emission + CFP emission)] in each particle (Figure 2A, right panels). We observed two major groups: virions colored in the green-blue spectrum (low FRET ratio, white arrowheads) and virions colored in the red spectrum (high FRET ratio, yellow arrowheads). Based on our construct design, we hypothesized that CFP and YFP are located next to each other in immature virions and have a high FRET ratio. On the other hand, CFP and YFP are separated and dispersed in mature virions, leading to a reduction of FRET efficiency (low FRET ratio). Consistent with our hypothesis, we observed that most of the HIV-1 Gag-iFRETΔPro labeled virions appeared in the red spectrum (Figure 2A, bottom right panel). Accordingly, maturation capable virions labeled by HIV-1 Gag-iFRET appeared mostly in the green-blue spectrum mixed with some particles maintaining a high FRET ratio (Figure 2A, top right panel). In other words, based on the FRET ratio view, the HIV-1 Gag-iFRETΔPro virion population comprises solely immature particles, while HIV-1 Gag-iFRET viruses revealed heterogeneous phenotypes of Gag maturation including both mature and immature cores.

We next quantified the proportion of mature and immature virions in the FRET labeled virus carrying the intact HIV-1 protease. The FRET ratio was calculated for each virion from the extracted FRET donor and acceptor signal intensities. FRET efficiencies were then plotted in histograms that reflected sample heterogeneity (Figure 2B and Supplementary Figure 1). As expected, the Normal Probability plot could fit a Gaussian distribution curve over the histogram plots of the HIV-1 Gag-iFRETΔPro population, which contained only immature virions (Supplementary Figure 1B). By comparison, the distribution of HIV-1 Gag-iFRET viruses did not fit a gaussian curve, consistent with the presence of a mixed virion population of mature and immature particles (Supplementary Figure 1A). Kernel density estimation is frequently used as a smoothening estimation function for non-normal distributions. Thus, we applied kernel density estimation curves and performed total density calculations in this analysis (Figure 2B). The Kernel density estimation curves of HIV-1 Gag-iFRET and -iFRETΔPro were overlapped after the adjustment of total particle counts (Figure 2C). We then measured the area occupied by the HIV-1 Gag-iFRET curve that merged with the HIV-1 Gag-iFRETΔPro curve and determined this as the proportion of immature virions out of the total corresponding HIV-1 Gag-iFRET area. We counted over 46,000 particles of HIV-1 Gag-iFRET and over 77,000 particles of HIV-1 Gag-iFRETΔPro labeled virions in three independent experiments. The overall proportion of immature virions in the HIV-1 Gag-iFRET population was 22.4% ± 2.4% calculated based on the 100% immaturity of HIV-1 Gag-iFRETΔPro (Figure 2D). We confirmed that these proportions were consistent with the rates determined by electron microscopy analysis in other reports (Burdick et al., 2020; Link et al., 2020) and ours (Figure 1E).

Taken together, we were able to visualize the maturation state of virions based on their FRET signal intensity using fluorescence microscopy and to quantify the proportion of immature virions with a rate comparable to that found by electron microscopy-based assays.

### Quantitative Assessment of Protease Inhibitor Activity Using the HIV-1 Gag-iFRET Single Virion Visualization System

**FIGURE 3:**
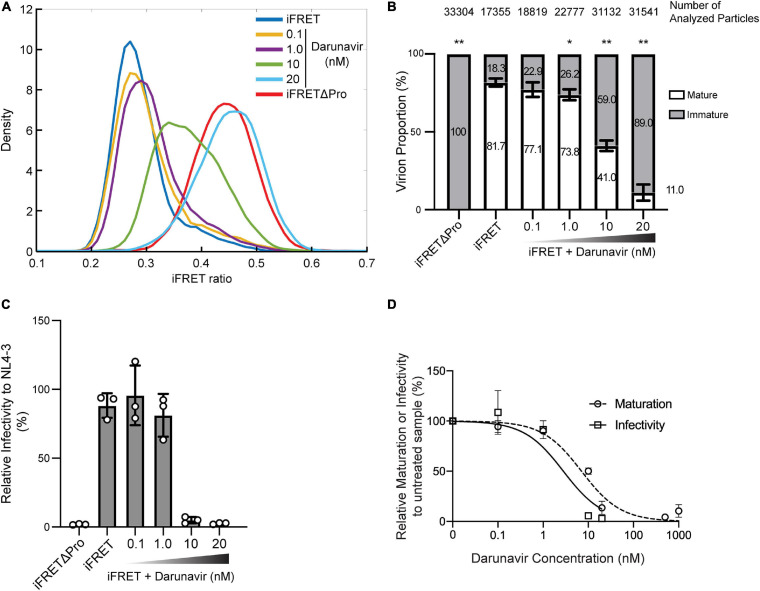
*In vitro* dose-dependent effect of a protease inhibitor detected with the HIV-1 Gag-iFRET system. **(A)** Kernel density estimation curves from a representative experiment of HIV-1 Gag-iFRET virions produced under treatment with a protease inhibitor, Darunavir, at four different concentrations (0.1, 1.0, 10, and 20 nM). **(B)** Quantification of mature and immature virion populations treated with Darunavir, as determined by overlapping kernel density estimation curves as described in Figure 2C. The stacked bar plot shows the average percentage of mature and immature virions in each untreated and treated population. Error bars indicate the standard deviation of three independent experiments. The size of the immature virion population in each Darunavir treated sample was compared to that of the untreated HIV-1 Gag-iFRET sample by paired *t*-test and significant differences are marked accordingly (**p < 0.01, *p < 0.05). **(C)** Single-round infectivity assays with TZM-bl cells were performed to determine the inhibitory activity of Darunavir at each tested concentration. The bar plot shows the infectivity (%) of HIV-1 Gag-iFRET virus with or without Darunavir treatment relative to the infectivity of the parental NL4-3 virus. Error bars indicate the standard deviation of three independent experiments. Statistical significance was calculated by Wilcoxon matched-pairs signed rank test compared to NL4-3 infectivity. **(D)** Dose-response curves for relative inhibition of maturation and infectivity at the tested Darunavir concentrations compared to the untreated sample. The EC_50_ and IC_50_ of Darunavir efficacy were calculated by maturation and infectivity rates at the range of 0.1–1000 nM and 0.1–20 nM concentrations of Darunavir, respectively.

In order to evaluate the applicability of the HIV-1 Gag-iFRET system, we sought to assess the efficacy of a protease inhibitor treatment by measuring the population of immature virions and correlating the results with the associated virus infectivity. For this purpose, we produced HIV-1 Gag-iFRET labeled virions in the absence or presence of Darunavir, a protease inhibitor used in the clinic to treat HIV-1 infection (De Meyer et al., 2005; Spagnuolo et al., 2018), and quantified the proportion of immature virions at four different concentrations. Darunavir treatment shifted the peak of the FRET ratio distribution to the right in a dose-dependent manner (Figure 3A). Virions produced by cells treated with the lowest concentration of Darunavir, 0.1 nM, were in the same FRET range as the non-treated control (Figure 3A, yellow line), while those treated with 20 nM Darunavir shifted to the iFRETΔPro FRET range (Figure 3A, light blue line). The peak of the virion population treated with 10 nM Darunavir was approximately halfway between the non-treated and immature controls (Figure 3A, green line).

We counted between 17,000 and 33,000 particles in total for each condition and quantified the proportion of immature virions with the same method described in Figure 2C (Figure 3B). The proportion of immature virions increased dose-dependently with Darunavir treatment from 22.9 to 89.0%. Correspondingly, we assessed the HIV-1 Gag-iFRET virus infectivity produced by cells treated with Darunavir (Figure 3C). Virus infectivity was not significantly affected by Darunavir concentrations up to 1.0 nM, whereas a drastic reduction in infectivity was observed at the 10 and 20 nM concentrations. According to the dose-response relationships of Darunavir concentration with virion maturation and virus infectivity (Figures 3B,C, respectively), the 50% effective concentration (EC_50_) against virion maturation was 7.0 nM, and the 50% inhibitory concentration (IC_50_) of virus infectivity was 2.8 nM (Figure 3D). This indicated that the drug concentration required to prevent virus maturation was approximately two-fold higher than that needed for antiviral effect. This suggests that some Darunavir treated viruses that completed maturation also lost infectivity.

In conclusion, the HIV-1 Gag-iFRET labeling strategy we described here was used to quantify the effects of a protease inhibitor on the maturation rate of HIV-1.

## Discussion

In this study, we set out to develop a FRET based fluorescence microscopy tool for a large-scale quantitative measurement of morphologically distinct mature and immature HIV-1 virus particles. Electron microscopy (EM) is a technique traditionally used for the structural determination of virion maturation (Lee and Gui, 2016). It remains a powerful method to identify morphological signatures in virions due to its high resolution. However, the proportions of mature virions measured by EM are usually assessed manually which leads to a large variation within the range of 80–99% of the total purified virions (De Marco et al., 2012; Keller et al., 2013; Mattei et al., 2015; Burdick et al., 2020; Link et al., 2020). Fluorescence microscopy on the other hand is expanding to comprise techniques capable of spatiotemporal analysis of the viral life cycle (Campbell and Hope, 2008; Francis and Melikyan, 2018). Hubner et al. (2007) has successfully produced infectious labeled virions by developing a fluorescently tagged HIV-1 construct, HIV Gag-iGFP. HIV Gag-iGFP has been used to track HIV-1 Gag protein through cellular compartments and visualize virological synapses in living cells (Hubner et al., 2009; Wang et al., 2019), but is unable to distinguish immature and mature virions. Although a number of mechanisms in the virus life cycle were elucidated by visualizing virus particles or components in the context of living cells, a fluorescence microscopy technique capable of showing the morphological transition from immature to mature state was still in need. In this work, we created a fluorescently distinguishable system based on the visualization of the Gag maturation status in virus particles. This system based on the FRET principle was achieved by inserting the optimized intracellular CFP-YFP FRET pair proteins (ECFPΔC11 and cp173Venus, respectively) (Nagai et al., 2004) between the MA and CA domains of Gag (HIV-1 Gag-iFRET; Figure 1A). The inserted CFP and YFP proteins were flanked by HIV-1 protease cleavage sites to allow the separation of the FRET pair proteins from Gag in the mature virion, which enabled us to differentiate mature and immature virions based on their FRET signal. High FRET intensities were observed in the immature virions generated by the HIV-1 Gag-iFRETΔPro construct due to the vicinity of the FRET donor and acceptor proteins in a single Gag molecule (Figure 2A, bottom panels). In our analyses, the histogram plots of FRET signal values derived from the HIV-1 Gag-iFRETΔPro population fitted a normal distribution curve (Figure 2B and Supplementary Figure 1B), indicating that the virus population consisted of a single phenotype of Gag protein with an immature conformation and also confirmed Gag-iFRETΔPro virions’ homogenous immature status. The CFP and YFP proteins inserted in the Gag polyprotein distributed within the viral particle once the two fluorescent proteins were cleaved apart during maturation, followed by FRET signal diminution (Figure 2A, upper panels). As not all protease-intact particles seemed to complete the maturation phase, the normal probability plot did not fit a Gaussian distribution (Supplementary Figure 1A). The HIV-1 Gag-iFRET virion population was heterogeneous and contained both mature and immature virions as confirmed by our TEM images (Figure 1E).

After having confirmed that HIV-1 Gag-iFRET successfully labeled infectious virions and that there was a measurable difference in the FRET signal emitted by mature and immature virions, we proceeded to quantify the proportion of immature virions. We calculated the overlapping area of HIV-1 Gag-iFRET with HIV-1 Gag-iFRETΔPro labeled virions to determine the proportion of immature virions out of the total HIV-1 Gag-iFRET viruses. As a result, nearly 20% of the HIV-1 Gag-iFRET virions were accounted to be immature (Figure 2). As we mentioned earlier, it has been previously reported that the frequency of immature virions ranges between 0.1 and 20% of the total HIV-1 particles counted in EM images (De Marco et al., 2012; Keller et al., 2013; Mattei et al., 2015; Burdick et al., 2020; Link et al., 2020). The frequency of the immature state measured through our FRET signal analysis was slightly higher (20%, Figure 2), but still in the range of previous EM reports (Burdick et al., 2020; Link et al., 2020) and consistent with our count (Figure 1E). Since we are not able to completely exclude false positive counts, as immature virions with a median FRET signal in HIV-1 Gag-iFRET overlapped with those with a lower signal in the protease deficient population, we believe our image analysis scheme has been optimized at this point. Taken together, immature virion quantification using HIV-1 Gag-iFRET yielded reproducible results over multiple experiments with the great advantage of being capable of large-scale virion quantification through semi-automated image processing.

A potential application of the HIV-1 Gag-iFRET system is live-cell imaging to study HIV-1 release at the budding site. Live-cell microscopy using GFP-tagged CA or other fluorescent molecules has provided invaluable information on the behavior of virus components, particularly the localization and various functions of the capsid (Hubner et al., 2009; Burdick et al., 2020; Zurnic Bonisch et al., 2020), and FRET has been used to measure the duration of virion assembly at the plasma membrane (Jouvenet et al., 2008). Hu’s group investigated the behavior of viral RNA in fluorescence imaging experiments using RNA-binding proteins that specifically recognize stem-loop sequences engineered into the viral genome (Chen et al., 2009) and revealed that only a portion of the HIV-1 RNAs that reach the plasma membrane became associated with viral protein complexes (Sardo et al., 2015). HIV Gag-iGFP was used in live-cell imaging to show virion trafficking during virological synapses (Hubner et al., 2009). Thus, the combination of RNA labeling techniques with the HIV-1 Gag-iFRET system would provide a unique method in this context to elucidate the dynamics of Gag-viral RNA release from the budding site into progeny virions. Moreover, viral assembly appears to be cell-type dependent (Ono and Freed, 2004), and virions are assembled and released in viral-containing compartments (VCCs) in macrophages, beyond the reach of antivirals or antibodies, and from where cell-to-cell infection can occur unhindered (Pelchen-Matthews et al., 2003; Sharova et al., 2005; Gousset et al., 2008; Groot et al., 2008; Chu et al., 2012; Inlora et al., 2016). HIV-1 Gag-iFRET could be used in live-cell imaging to localize and visualize maturation in various cellular compartments in different cell-type settings. This would circumvent the limitation of some studies in which HIV-1 components are found in VCCs after endocytosis or phagocytosis of the newly released particles (Jouvenet et al., 2006). Together with the large-scale quantification approach to image analysis, our system can provide new and reliable insights into this fundamental step of the HIV-1 life cycle.

To further evaluate the HIV-1 Gag-iFRET quantitative potential, we tested its applicability to antiretroviral drug assessment. For this purpose, we examined the sensitivity of the HIV-1 Gag-iFRET system in detecting changes in the immature HIV-1 virion population after treatment with the protease inhibitor, Darunavir. A dose-dependent increase of the FRET signals associated with escalation of the immature virion population was observed (Figure 3A). According to the single round infectivity assays in TZM-bl cells, the 50% inhibitory concentration (IC_50_) of Darunavir was 2.8 nM (0.88–8.3 nM in 95% Confidence Interval) (Figure 3D), which was in the range of previous reports (1–5 nM) (De Meyer et al., 2005). On the other hand, the 50% effective concentration (EC_50_) of Darunavir as a protease inhibitor calculated by the frequency of immature virions in our FRET labeling system was 7.0 nM (3.9–12.1 nM in 95% Confidence Interval). We observed that more than double the concentration of IC_50_ is required to inhibit maturation in 50% of the virions (Figure 3D). Despite the shift between the two assays being only twofold, this remains an interesting observation showing that using only infectivity assays to determine the specific effect of protease inhibitors on maturation might be insufficient. It has been previously suggested that protease inhibitors including Darunavir also block virus entry, reverse transcription, and integration steps (Rabi et al., 2013). Thus, it stands to reason that Darunavir’s IC_50_ is different from its EC_50_ in our calculations. According to this discordance, it could be inferred that approximately half of the particles inactivated by Darunavir still completed maturation. Further studies are needed to elucidate these observations.

It is possible that defective viruses can still produce antigens and virus-like particles that could undergo the maturation process. In this regard, it has been shown that defective viruses play a role in preferentially activating CD4 T cells for productive HIV-1 replication, and in providing a large pool of HIV-1 epitopes that continuously stimulate CD4 T cells with different antigen specificity (Finzi et al., 2006). Our results implied that they might do so in a mature conformation. In support of this idea, studies that looked at the possibility of using defective virions that can only produce virus-like particles for immunization purposes found that the immature morphology enhanced particles’ immunogenicity including stimulation of T cell responses, such as IFN-γ production, and eliciting Env targeting antibody production (Alvarez-Fernandez et al., 2012; Gonelli et al., 2019). This further emphasizes the importance of maturation for both treatment and prevention of HIV-1 infection. In addition, more than 95% of proviruses in the peripheral blood are defective in people living with HIV-1 on combination antiretroviral therapy (Ho et al., 2013; Bruner et al., 2016). Defective proviruses have recently been reported to encode novel unspliced forms of HIV-1 RNA transcripts with competent open reading frames and subsequent structural protein expression that may lead to persistent immune activation by triggering both innate and adaptive immunity (Ho et al., 2013; Imamichi et al., 2016). As the majority of defective proviruses have large internal deletions but preserve intact gag or gag-pol sequences, it is possible that defective proviruses form extracellular virus-like particles that activate immune responses. Thus, the maturation status of extracellular defective viruses becomes increasingly important for estimating their potential immunogenicity and our novel FRET labeling system would be suitable for investigating this matter. In addition, the viral integrase has been also shown to be necessary for correct HIV-1 maturation (Fontana et al., 2015; Kessl et al., 2016; Elliott and Kutluay, 2020) and its mechanism of action could also be explored using HIV-1 Gag-iFRET.

## Conclusion

The HIV-1 Gag-iFRET system, together with the semi-automated, unbiased imaging and analysis strategy provided in this work are a new, powerful addition to the virological and biological molecular tools set. While the current major focus for HIV-1 functional cure strategy is toward reactivating latently infected cells and their elimination, biological activity and pathogenesis of defective proviruses are drawing attention as another potential obstacle to a functional cure. HIV-1 Gag-iFRET can be used to more thoroughly investigate maturation, when viruses acquire their infectivity and immunogenicity. Elucidation of the space-time frame of maturation may reveal therapeutic windows and help broaden our antiviral arsenal.

## Data Availability Statement

The original contributions presented in the study are included in the article/Supplementary Material, further inquiries can be directed to the corresponding author/s.

## Author Contributions

AS, LS, and TI performed the experiments. KH, KS, AT-K, and TI designed the study. AS, HF, HM, KS, and TI analyzed the data. AS, LS, and TI wrote the manuscript. AS, AT-K, and TI contributed to financial assistance. All the authors contributed to the article and approved the submitted version.

## Conflict of Interest

The authors declare that the research was conducted in the absence of any commercial or financial relationships that could be construed as a potential conflict of interest.

## Supplementary Material

Supplementary Figure 1 Gaussian fitting and normal distribution probability calculation for HIV-1 Gag-iFRET and -iFRETΔPro virion populations. A Gaussian (normal) distribution curve (red line) was fitted to the histograms shown in **(A)** Figure 2B or **(B)** Figure 2C. The normal probability plot assessed the Gaussian distribution of the representative data set.

Supplementary Table 1 HIV-1 Gag-iFRET insert sequence.

## References

[B1] Adachi A. Ono N. Sakai H. Ogawa K. Shibata R. Kiyomasu T. (1991). Generation and characterization of the human immunodeficiency virus type 1 mutants. *Arch. Virol.* 117 45–58. 10.1007/bf01310491 1706590

[B2] Alvarez-Fernandez C. Crespo Guardo A. Garcia-Perez J. Garcia F. Blanco J. Escriba-Garcia L. (2012). Generation and characterization of a defective HIV-1 Virus as an immunogen for a therapeutic vaccine. *PLoS One* 7:e48848. 10.1371/journal.pone.0048848 23144996 PMC3492255

[B3] Bajar B. T. Wang E. S. Zhang S. Lin M. Z. Chu J. (2016). A Guide to fluorescent protein FRET pairs. *Sensors (Basel)* 16:1488. 10.3390/s16091488 27649177 PMC5038762

[B4] Bruner K. M. Murray A. J. Pollack R. A. Soliman M. G. Laskey S. B. Capoferri A. A. (2016). Defective proviruses rapidly accumulate during acute HIV-1 infection. *Nat. Med.* 22 1043–1049. 10.1038/nm.4156 27500724 PMC5014606

[B5] Burdick R. C. Li C. Munshi M. Rawson J. M. O. Nagashima K. Hu W. S. (2020). HIV-1 uncoats in the nucleus near sites of integration. *Proc. Natl. Acad. Sci. U.S.A.* 117 5486–5493. 10.1073/pnas.1920631117 32094182 PMC7071919

[B6] Campbell E. M. Hope T. J. (2008). Live cell imaging of the HIV-1 life cycle. *Trends Microbiol.* 16 580–587. 10.1016/j.tim.2008.09.006 18977142 PMC3209483

[B7] Castilla J. Del Romero J. Hernando V. Marincovich B. Garcia S. Rodriguez C. (2005). Effectiveness of highly active antiretroviral therapy in reducing heterosexual transmission of HIV. *J. Acquir. Immune Defic. Syndr.* 40 96–101. 10.1097/01.qai.0000157389.78374.45 16123689

[B8] Centers for Disease Control and Prevention (CDC) (2006). HHS-CDC news: the global HIV/AIDS pandemic, 2006. *Ann. Pharmacother.* 40:1708. 10.1345/aph.1n117 16912242

[B9] Chen J. Nikolaitchik O. Singh J. Wright A. Bencsics C. E. Coffin J. M. (2009). High efficiency of HIV-1 genomic RNA packaging and heterozygote formation revealed by single virion analysis. *Proc. Natl. Acad. Sci. U.S.A.* 106 13535–13540. 10.1073/pnas.0906822106 19628694 PMC2714765

[B10] Chiu T. Y. Yang D. M. (2012). Intracellular Pb2+ content monitoring using a protein-based Pb2+ indicator. *Toxicol. Sci.* 126 436–445. 10.1093/toxsci/kfs007 22240981

[B11] Chu H. Wang J. J. Qi M. Yoon J. J. Wen X. Chen X. (2012). The intracellular virus-containing compartments in primary human macrophages are largely inaccessible to antibodies and small molecules. *PLoS One* 7:e35297. 10.1371/journal.pone.0035297 22567100 PMC3342280

[B12] Chun T. W. Stuyver L. Mizell S. B. Ehler L. A. Mican J. A. Baseler M. (1997). Presence of an inducible HIV-1 latent reservoir during highly active antiretroviral therapy. *Proc. Natl. Acad. Sci. U.S.A.* 94 13193–13197. 10.1073/pnas.94.24.13193 9371822 PMC24285

[B13] Colby D. J. Trautmann L. Pinyakorn S. Leyre L. Pagliuzza A. Kroon E. (2018). Rapid HIV RNA rebound after antiretroviral treatment interruption in persons durably suppressed in Fiebig I acute HIV infection. *Nat. Med.* 24 923–926. 10.1038/s41591-018-0026-6 29892063 PMC6092240

[B14] Daar E. S. Bai J. Hausner M. A. Majchrowicz M. Tamaddon M. Giorgi J. V. (1998). Acute HIV syndrome after discontinuation of antiretroviral therapy in a patient treated before seroconversion. *Ann. Intern. Med.* 128 827–829. 10.7326/0003-4819-128-10-199805150-00005 9599194

[B15] Davidoff C. Payne R. J. Willis S. H. Doranz B. J. Rucker J. B. (2012). Maturation of the Gag core decreases the stability of retroviral lipid membranes. *Virology* 433 401–409. 10.1016/j.virol.2012.08.023 22995186 PMC3848701

[B16] De Marco A. Heuser A. M. Glass B. Krausslich H. G. Muller B. Briggs J. A. (2012). Role of the SP2 domain and its proteolytic cleavage in HIV-1 structural maturation and infectivity. *J. Virol.* 86 13708–13716. 10.1128/jvi.01704-12 23055560 PMC3503038

[B17] De Meyer S. Azijn H. Surleraux D. Jochmans D. Tahri A. Pauwels R. (2005). TMC114, a novel human immunodeficiency virus type 1 protease inhibitor active against protease inhibitor-resistant viruses, including a broad range of clinical isolates. *Antimicrob. Agents Chemother.* 49 2314–2321. 10.1128/aac.49.6.2314-2321.2005 15917527 PMC1140553

[B18] De Rocquigny H. El Meshri S. E. Richert L. Didier P. Darlix J. L. Mely Y. (2014). Role of the nucleocapsid region in HIV-1 Gag assembly as investigated by quantitative fluorescence-based microscopy. *Virus Res.* 193 78–88. 10.1016/j.virusres.2014.06.009 25016037

[B19] Elliott J. L. Kutluay S. B. (2020). Going beyond Integration: the emerging role of HIV-1 integrase in virion morphogenesis. *Viruses* 12:1005. 10.3390/v12091005 32916894 PMC7551943

[B20] Finzi D. Blankson J. Siliciano J. D. Margolick J. B. Chadwick K. Pierson T. (1999). Latent infection of CD4+ T cells provides a mechanism for lifelong persistence of HIV-1, even in patients on effective combination therapy. *Nat. Med.* 5 512–517. 10.1038/8394 10229227

[B21] Finzi D. Hermankova M. Pierson T. Carruth L. M. Buck C. Chaisson R. E. (1997). Identification of a reservoir for HIV-1 in patients on highly active antiretroviral therapy. *Science* 278 1295–1300. 10.1126/science.278.5341.1295 9360927

[B22] Finzi D. Plaeger S. F. Dieffenbach C. W. (2006). Defective virus drives human immunodeficiency virus infection, persistence, and pathogenesis. *Clin. Vaccine Immunol.* 13 715–721. 10.1128/cvi.00052-06 16829607 PMC1489566

[B23] Fontana J. Jurado K. A. Cheng N. Ly N. L. Fuchs J. R. Gorelick R. J. (2015). Distribution and redistribution of HIV-1 nucleocapsid protein in immature, mature, and integrase-inhibited virions: a role for integrase in maturation. *J. Virol.* 89 9765–9780. 10.1128/jvi.01522-15 26178982 PMC4577894

[B24] Forshey B. M. Von Schwedler U. Sundquist W. I. Aiken C. (2002). Formation of a human immunodeficiency virus type 1 core of optimal stability is crucial for viral replication. *J. Virol.* 76 5667–5677. 10.1128/jvi.76.11.5667-5677.2002 11991995 PMC137032

[B25] Francis A. C. Melikyan G. B. (2018). Live-cell imaging of early steps of single HIV-1 infection. *Viruses* 10:275. 10.3390/v10050275 29783762 PMC5977268

[B26] Freed E. O. (2015). HIV-1 assembly, release and maturation. *Nat. Rev. Microbiol.* 13 484–496. 10.1038/nrmicro3490 26119571 PMC6936268

[B27] Fukuda H. Li S. Sardo L. Smith J. L. Yamashita K. Sarca A. D. (2019). Structural determinants of the APOBEC3G N-terminal domain for HIV-1 RNA association. *Front. Cell Infect. Microbiol.* 9:129. 10.3389/fcimb.2019.00129 PMC6536580 31165049

[B28] Gao D. Wu J. Wu Y. T. Du F. Aroh C. Yan N. (2013). Cyclic GMP-AMP synthase is an innate immune sensor of HIV and other retroviruses. *Science* 341 903–906. 10.1126/science.1240933 23929945 PMC3860819

[B29] Gao F. Morrison S. G. Robertson D. L. Thornton C. L. Craig S. Karlsson G. (1996). Molecular cloning and analysis of functional envelope genes from human immunodeficiency virus type 1 sequence subtypes A through G. The WHO and NIAID networks for HIV isolation and characterization. *J. Virol.* 70 1651–1667. 10.1128/jvi.70.3.1651-1667.1996 8627686 PMC189989

[B30] Gonelli C. A. Khoury G. Center R. J. Purcell D. F. J. (2019). HIV-1-based Virus-like particles that morphologically resemble mature, infectious HIV-1 virions. *Viruses* 11:507. 10.3390/v11060507 31159488 PMC6630479

[B31] Gousset K. Ablan S. D. Coren L. V. Ono A. Soheilian F. Nagashima K. (2008). Real-time visualization of HIV-1 GAG trafficking in infected macrophages. *PLoS Pathog.* 4:e1000015. 10.1371/journal.ppat.1000015 18369466 PMC2267008

[B32] Groot F. Welsch S. Sattentau Q. J. (2008). Efficient HIV-1 transmission from macrophages to T cells across transient virological synapses. *Blood* 111 4660–4663. 10.1182/blood-2007-12-130070 18296630

[B33] Hassan J. Browne K. De Gascun C. (2016). HIV-1 in monocytes and macrophages: an overlooked reservoir? *Viral. Immunol.* 29 532–533. 10.1089/vim.2016.0091 27564791

[B34] Heim R. Tsien R. Y. (1996). Engineering green fluorescent protein for improved brightness, longer wavelengths and fluorescence resonance energy transfer. *Curr. Biol.* 6 178–182. 10.1016/s0960-9822(02)00450-5 8673464

[B35] Henrich T. J. Hatano H. Bacon O. Hogan L. E. Rutishauser R. Hill A. (2017). HIV-1 persistence following extremely early initiation of antiretroviral therapy (ART) during acute HIV-1 infection: an observational study. *PLoS Med.* 14:e1002417. 10.1371/journal.pmed.1002417 PMC5675377 29112956

[B36] Hiener B. Horsburgh B. A. Eden J. S. Barton K. Schlub T. E. Lee E. (2017). Identification of genetically intact HIV-1 proviruses in specific CD4(+) T cells from effectively treated participants. *Cell Rep.* 21 813–822. 10.1016/j.celrep.2017.09.081 29045846 PMC5960642

[B37] Ho Y. C. Shan L. Hosmane N. N. Wang J. Laskey S. B. Rosenbloom D. I. (2013). Replication-competent noninduced proviruses in the latent reservoir increase barrier to HIV-1 cure. *Cell* 155 540–551. 10.1016/j.cell.2013.09.020 24243014 PMC3896327

[B38] Holkmann Olsen C. Mocroft A. Kirk O. Vella S. Blaxhult A. Clumeck N. (2007). Interruption of combination antiretroviral therapy and risk of clinical disease progression to AIDS or death. *HIV Med.* 8 96–104. 10.1111/j.1468-1293.2007.00436.x 17352766

[B39] Hubner W. Chen B. K. (2006). Inhibition of viral assembly in murine cells by HIV-1 matrix. *Virology* 352 27–38. 10.1016/j.virol.2006.04.024 16750235

[B40] Hubner W. Chen P. Del Portillo A. Liu Y. Gordon R. E. Chen B. K. (2007). Sequence of human immunodeficiency virus type 1 (HIV-1) Gag localization and oligomerization monitored with live confocal imaging of a replication-competent, fluorescently tagged HIV-1. *J. Virol.* 81 12596–12607. 10.1128/jvi.01088-07 17728233 PMC2168995

[B41] Hubner W. Mcnerney G. P. Chen P. Dale B. M. Gordon R. E. Chuang F. Y. (2009). Quantitative 3D video microscopy of HIV transfer across T cell virological synapses. *Science* 323 1743–1747. 10.1126/science.1167525 19325119 PMC2756521

[B42] Imamichi H. Dewar R. L. Adelsberger J. W. Rehm C. A. O’doherty U. Paxinos E. E. (2016). Defective HIV-1 proviruses produce novel protein-coding RNA species in HIV-infected patients on combination antiretroviral therapy. *Proc. Natl. Acad. Sci. U.S.A.* 113 8783–8788. 10.1073/pnas.1609057113 27432972 PMC4978246

[B43] Imamichi H. Smith M. Adelsberger J. W. Izumi T. Scrimieri F. Sherman B. T. (2020). Defective HIV-1 proviruses produce viral proteins. *Proc. Natl. Acad. Sci. U.S.A.* 117 3704–3710. 10.1073/pnas.1917876117 32029589 PMC7035625

[B44] Inlora J. Chukkapalli V. Bedi S. Ono A. (2016). Molecular determinants directing HIV-1 gag assembly to virus-containing compartments in primary macrophages. *J. Virol.* 90 8509–8519. 10.1128/jvi.01004-16 27440886 PMC5021390

[B45] Jouvenet N. Bieniasz P. D. Simon S. M. (2008). Imaging the biogenesis of individual HIV-1 virions in live cells. *Nature* 454 236–240. 10.1038/nature06998 18500329 PMC2708942

[B46] Jouvenet N. Neil S. J. Bess C. Johnson M. C. Virgen C. A. Simon S. M. (2006). Plasma membrane is the site of productive HIV-1 particle assembly. *PLoS Biol.* 4:e435. 10.1371/journal.pbio.0040435 17147474 PMC1750931

[B47] Keller P. W. Huang R. K. England M. R. Waki K. Cheng N. Heymann J. B. (2013). A two-pronged structural analysis of retroviral maturation indicates that core formation proceeds by a disassembly-reassembly pathway rather than a displacive transition. *J. Virol.* 87 13655–13664. 10.1128/jvi.01408-13 24109217 PMC3838239

[B48] Kessl J. J. Kutluay S. B. Townsend D. Rebensburg S. Slaughter A. Larue R. C. (2016). HIV-1 integrase binds the Viral RNA genome and is essential during virion morphogenesis. *Cell* 166 1257–1268.e1212. 27565348 10.1016/j.cell.2016.07.044 PMC5003418

[B49] Kitahata M. M. Gange S. J. Abraham A. G. Merriman B. Saag M. S. Justice A. C. (2009). Effect of early versus deferred antiretroviral therapy for HIV on survival. *N. Engl. J. Med.* 360 1815–1826. 19339714 10.1056/NEJMoa0807252 PMC2854555

[B50] Kousignian I. Abgrall S. Grabar S. Mahamat A. Teicher E. Rouveix E. (2008). Maintaining antiretroviral therapy reduces the risk of AIDS-defining events in patients with uncontrolled viral replication and profound immunodeficiency. *Clin. Infect. Dis.* 46 296–304. 10.1086/524753 18171266

[B51] Kremers G. J. Goedhart J. Van Munster E. B. Gadella T. W. Jr. (2006). Cyan and yellow super fluorescent proteins with improved brightness, protein folding, and FRET Forster radius. *Biochemistry* 45 6570–6580. 10.1021/bi0516273 16716067

[B52] Lee K. K. Gui L. (2016). Dissecting virus infectious cycles by cryo-electron microscopy. *PLoS Pathog.* 12:e1005625. 10.1371/journal.ppat.1005625 27362353 PMC4928862

[B53] Link J. O. Rhee M. S. Tse W. C. Zheng J. Somoza J. R. Rowe W. (2020). Clinical targeting of HIV capsid protein with a long-acting small molecule. *Nature* 584 614–618. 32612233 10.1038/s41586-020-2443-1 PMC8188729

[B54] Mattei S. Flemming A. Anders-Osswein M. Krausslich H. G. Briggs J. A. Muller B. (2015). RNA and nucleocapsid are dispensable for mature HIV-1 capsid assembly. *J. Virol.* 89 9739–9747. 10.1128/jvi.00750-15 26178992 PMC4577892

[B55] Mattei S. Tan A. Glass B. Muller B. Krausslich H. G. Briggs J.a.G (2018). High-resolution structures of HIV-1 Gag cleavage mutants determine structural switch for virus maturation. *Proc. Natl. Acad. Sci. U.S.A.* 115 E9401–E9410. 30217893 10.1073/pnas.1811237115 PMC6176557

[B56] Muller B. Anders M. Reinstein J. (2014). In vitro analysis of human immunodeficiency virus particle dissociation: gag proteolytic processing influences dissociation kinetics. *PLoS One* 9:e99504. 10.1371/journal.pone.0099504 24915417 PMC4051761

[B57] Nagai T. Yamada S. Tominaga T. Ichikawa M. Miyawaki A. (2004). Expanded dynamic range of fluorescent indicators for Ca(2+) by circularly permuted yellow fluorescent proteins. *Proc. Natl. Acad. Sci. U.S.A.* 101 10554–10559. 10.1073/pnas.0400417101 15247428 PMC490022

[B58] Novikova M. Zhang Y. Freed E. O. Peng K. (2019). Multiple roles of HIV-1 capsid during the virus replication cycle. *Virol. Sin.* 34 119–134. 10.1007/s12250-019-00095-3 31028522 PMC6513821

[B59] Ono A. Freed E. O. (2004). Cell-type-dependent targeting of human immunodeficiency virus type 1 assembly to the plasma membrane and the multivesicular body. *J. Virol.* 78 1552–1563. 10.1128/jvi.78.3.1552-1563.2004 14722309 PMC321403

[B60] Pelchen-Matthews A. Kramer B. Marsh M. (2003). Infectious HIV-1 assembles in late endosomes in primary macrophages. *J. Cell Biol.* 162 443–455. 10.1083/jcb.200304008 12885763 PMC2172706

[B61] Pornillos O. Ganser-Pornillos B. K. (2019). Maturation of retroviruses. *Curr. Opin. Virol.* 36 47–55. 10.1016/j.coviro.2019.05.004 31185449 PMC6730672

[B62] Preus S. Wilhelmsson L. M. (2012). Advances in quantitative FRET-based methods for studying nucleic acids. *Chembiochem* 13 1990–2001. 10.1002/cbic.201200400 22936620

[B63] Rabi S. A. Laird G. M. Durand C. M. Laskey S. Shan L. Bailey J. R. (2013). Multi-step inhibition explains HIV-1 protease inhibitor pharmacodynamics and resistance. *J. Clin. Invest.* 123 3848–3860. 10.1172/jci67399 23979165 PMC4381280

[B64] Rankovic S. Varadarajan J. Ramalho R. Aiken C. Rousso I. (2017). Reverse transcription mechanically initiates HIV-1 capsid disassembly. *J. Virol.* 91:e00289-17. 10.1128/JVI.00289-17 PMC5446659 28381579

[B65] Sardo L. Hatch S. C. Chen J. Nikolaitchik O. Burdick R. C. Chen D. (2015). Dynamics of HIV-1 RNA near the plasma membrane during virus assembly. *J. Virol.* 89 10832–10840. 10.1128/jvi.01146-15 26292321 PMC4621114

[B66] Sekar R. B. Periasamy A. (2003). Fluorescence resonance energy transfer (FRET) microscopy imaging of live cell protein localizations. *J. Cell Biol.* 160 629–633. 10.1083/jcb.200210140 12615908 PMC2173363

[B67] Sharova N. Swingler C. Sharkey M. Stevenson M. (2005). Macrophages archive HIV-1 virions for dissemination in trans. *EMBO J.* 24 2481–2489. 10.1038/sj.emboj.7600707 15920469 PMC1173148

[B68] Siddiqui M. A. Saito A. Halambage U. D. Ferhadian D. Fischer D. K. Francis A. C. (2019). A novel phenotype links HIV-1 capsid stability to cGAS-mediated DNA sensing. *J. Virol.* 93:e00706-19. 10.1128/JVI.00706-19 PMC6675898 31167922

[B69] Siliciano J. D. Kajdas J. Finzi D. Quinn T. C. Chadwick K. Margolick J. B. (2003). Long-term follow-up studies confirm the stability of the latent reservoir for HIV-1 in resting CD4+ T cells. *Nat. Med.* 9 727–728. 10.1038/nm880 12754504

[B70] Sood C. Francis A. C. Desai T. M. Melikyan G. B. (2017). An improved labeling strategy enables automated detection of single-virus fusion and assessment of HIV-1 protease activity in single virions. *J. Biol. Chem.* 292 20196–20207. 10.1074/jbc.m117.818088 29046351 PMC5724006

[B71] Spagnuolo V. Castagna A. Lazzarin A. (2018). Darunavir for the treatment of HIV infection. *Expert Opin. Pharmacother.* 19 1149–1163. 29913082 10.1080/14656566.2018.1484901

[B72] Wang L. Izadmehr S. Kamau E. Kong X. P. Chen B. K. (2019). Sequential trafficking of Env and Gag to HIV-1 T cell virological synapses revealed by live imaging. *Retrovirology* 16:2. 10.1186/s12977-019-0464-3 PMC6334456 30646921

[B73] Whitney J. B. Hill A. L. Sanisetty S. Penaloza-Macmaster P. Liu J. Shetty M. (2014). Rapid seeding of the viral reservoir prior to SIV viraemia in rhesus monkeys. *Nature* 512 74–77. 10.1038/nature13594 25042999 PMC4126858

[B74] Wong M. E. Jaworowski A. Hearps A. C. (2019). The HIV Reservoir in monocytes and macrophages. *Front. Immunol.* 10:1435. 10.3389/fimmu.2019.01435 PMC6607932 31297114

[B75] Zhang L. Chung C. Hu B. S. He T. Guo Y. Kim A. J. (2000). Genetic characterization of rebounding HIV-1 after cessation of highly active antiretroviral therapy. *J. Clin. Invest.* 106 839–845. 10.1172/jci10565 11018071 PMC517816

[B76] Zurnic Bonisch I. Dirix L. Lemmens V. Borrenberghs D. De Wit F. Vernaillen F. (2020). Capsid-labelled HIV to investigate the role of capsid during nuclear import and integration. *J. Virol.* 94:e01024-19. 10.1128/JVI.01024-19 PMC7081887 31941774

