# Supplementary figures and images for: FRET-Based Detection and Quantification of HIV-1 Virion Maturation
Source: Front Microbiol. 2021 Mar 9;12:647452. doi: 10.3389/fmicb.2021.647452 (PMC7985248; doi:10.3389/fmicb.2021.647452)

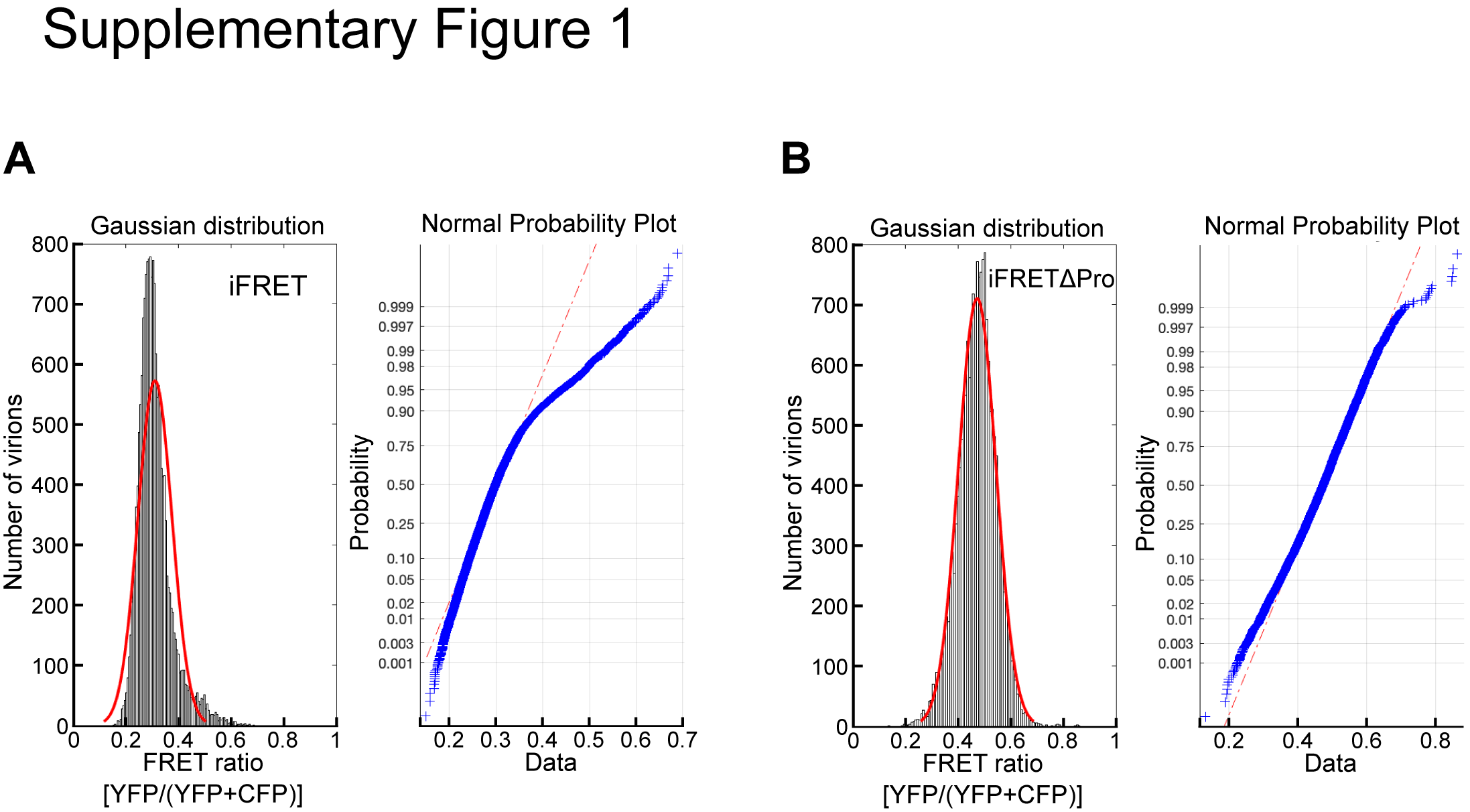

Supplement: Supplementary Figure 1 — Gaussian fitting and normal distribution probability calculation for HIV-1 Gag-iFRET and -iFRETΔPro virion populations. A Gaussian (normal) distribution curve (red line) was fitted to the histograms shown in (A) Figure 2B or (B) Figure 2C. The normal probability plot assessed the Gaussian distribution of the representative data set. [file Image_1.TIF]
