# Supplementary material for: FRET-Based Detection and Quantification of HIV-1 Virion Maturation
Source: Front Microbiol. 2021 Mar 9;12:647452. doi: 10.3389/fmicb.2021.647452 (PMC7985248; doi:10.3389/fmicb.2021.647452)
Supplement: Supplementary Table 1 — HIV-1 Gag-iFRET insert sequence. [file Table_1.docx]

| **Supplemental Table 1.** HIV-1 Gag-iFRET insert sequence | |
| --- | --- |
|  |  |
| Insert outline | Sequence |
| BssHII - 5’-LTR (truncated)- MA – Protease cleavage sequence - ECFP∆C11 - Protease cleavage sequence - cp173Venus - Protease cleavage sequence - 5’ end of CA - SphI | CGCGCACGGCAAGAGGCGAGGGGCGGCGACTGGTGAGTACGCCAAAAATTTTGACTAGCGGAGGCTAGAAGGAGAGAGATGGGTGCGAGAGCGTCGGTATTAAGCGGGGGAGAATTAGATAAATGGGAAAAAATTCGGTTAAGGCCAGGGGGAAAGAAACAATATAAACTAAAACATATAGTATGGGCAAGCAGGGAGCTAGAACGATTCGCAGTTAATCCTGGCCTTTTAGAGACATCAGAAGGCTGTAGACAAATACTGGGACAGCTACAACCATCCCTTCAGACAGGATCAGAAGAACTTAGATCATTATATAATACAATAGCAGTCCTCTATTGTGTGCATCAAAGGATAGATGTAAAAGACACCAAGGAAGCCTTAGATAAGATAGAGGAAGAGCAAAACAAAAGTAAGAAAAAGGCACAGCAAGCAGCAGCTGACACAGGAAACAACAGCCAGGTCtcgcagaactatccaattgtacaaATGGTGAGCAAGGGCGAGGAGCTGTTCACCGGGGTGGTGCCCATCCTGGTCGAGCTGGACGGCGACGTAAACGGCCACAAGTTCAGCGTGTCCGGCGAGGGCGAGGGCGATGCCACCTACGGCAAGCTGACCCTGAAGTTCATCTGCACCACCGGCAAGCTGCCCGTGCCCTGGCCCACCCTCGTGACCACCCTGACCTGGGGCGTGCAGTGCTTCAGCCGCTACCCCGACCACATGAAGCAGCACGACTTCTTCAAGTCCGCCATGCCCGAAGGCTACGTCCAGGAGCGCACCATCTTCTTCAAGGACGACGGCAACTACAAGACCCGCGCCGAGGTGAAGTTCGAGGGCGACACCCTGGTGAACCGCATCGAGCTGAAGGGCATCGACTTCAAGGAGGACGGCAACATCCTGGGGCACAAGCTGGAGTACAACTACATCAGCCACAACGTCTATATCACCGCCGACAAGCAGAAGAACGGCATCAAGGCCAACTTCAAGATCCGCCACAACATCGAGGACGGCAGCGTGCAGCTCGCCGACCACTACCAGCAGAACACCCCCATCGGCGACGGCCCCGTGCTGCTGCCCGACAACCACTACCTGAGCACCCAGTCCGCCCTGAGCAAAGACCCCAACGAGAAGCGCGATCACATGGTCCTGCTGGAGTTCGTGACCGCCGCCGGGtcgcagaactatccaattgtacaaGACGGCggcGTGCAGCTCGCCGACCACTACCAGCAGAACACCCCCATCGGCGACGGCCCCGTGCTGCTGCCCGACAACCACTACCTGAGCTACCAGTCCGCCCTGAGCAAAGACCCCAACGAGAAGCGCGATCACATGGTCCTGCTGGAGTTCGTGACCGCCGCCGGGATCACTCTCGGCATGGACGAGCTGTACAAGggcggctccggcggcATGGTGAGCAAGGGCGAGGAGCTGTTCACCGGGGTGGTGCCCATCCTGGTCGAGCTGGACGGCGACGTAAACGGCCACAAGTTCAGCGTGTCCGGCGAGGGCGAGGGCGATGCCACCTACGGCAAGCTGACCCTGAAGctgATCTGCACCACCGGCAAGCTGCCCGTGCCCTGGCCCACCCTCGTGACCACCctgGGCTACGGCCTGCAGTGCTTCGCCCGCTACCCCGACCACATGAAGCAGCACGACTTCTTCAAGTCCGCCATGCCCGAAGGCTACGTCCAGGAGCGCACCATCTTCTTCAAGGACGACGGCAACTACAAGACCCGCGCCGAGGTGAAGTTCGAGGGCGACACCCTGGTGAACCGCATCGAGCTGAAGGGCATCGACTTCAAGGAGGACGGCAACATCCTGGGGCACAAGCTGGAGTACAACTACAACAGCCACAACGTCTATATCaccGCCGACAAGCAGAAGAACGGCATCAAGgccAACTTCAAGATCCGCCACAACATCGAGAGCCAAAATTACCCTATAGTGCAGAACCTCCAGGGGCAAATGGTACATCAGGCCATATCACCTAGAACTTTAAATGCATGGGTAAAAGTAGTAGAAGAGAAGGCTTTCAGCCCAGAAGTAATACCCATGTTTTCAGCATTATCAGAAGGAGCCACCCCACAAGATTTAAATACCATGCTAAACACAGTGGGGGGACATCAAGCAGCCATGCAAATGTTAAAAGAGACCATCAATGAGGAAGCTGCAGAATGGGATAGATTGCATCCAGTGCATG |
